# Years of life lost for 137 causes of death in Belgium by age, sex, and region, 2004-2018: Aline Scohy

**DOI:** 10.1093/eurpub/ckac129.622

**Published:** 2022-10-25

**Authors:** A Scohy, R De Pauw, V Gorasso, L Van den Borre, B Devleesschauwer

**Affiliations:** 1 Department of Epidemiology and Public Health, Sciensano, Brussels, Belgium; 2 Department of Rehabilitation Sciences, Ghent University, Ghent, Belgium; 3 Department of Public Health and Primary Care, Ghent University, Ghent, Belgium; 4 Interface Demography, Vrije Universiteit Brussel, Brussels, Belgium; 5 Department of Translational Physiology, Infectiology and Public Health, Ghent University, Ghent, Belgium

## Abstract

Information on years of life lost (YLLs) due to premature mortality is necessary to assess the fatal impact of disease, crucial for the calculation of Belgian disability-adjusted life years (DALYs). This study presents a novel method to redistribute cause of death data. Belgian cause of death data are obtained from Statistics Belgium (Statbel). After mapping the ICD-10 codes defining the underlying cause of death to the GBD cause list, we redistributed ill-defined deaths (IDDs) to specific causes using a four-step probabilistic redistribution process developed to fit the Belgian context: internal redistribution, redistribution using predefined ICD codes, redistribution using multiple causes of death data, and redistribution to all causes. Finally, we used the GBD 2019 standard life expectancy table to calculate the years of life lost at age of death. In Belgium, between 2004 and 2018, IDDs increased from 31% to 34% of all deaths, reflecting increases in the average age at death. The majority was redistributed using predefined ICD codes (13%), followed by the redistribution using multiple causes of death data (10-11%). The total number of YLLs decreased from 1.83 to 1.77 million. In 2018, the top causes of YLLs were ischemic heart disease and lung cancer with a share of 8.4% each, followed by dementia and cerebrovascular disease with a share of 5.5% each. All results are stratified by age, sex, region, and year, and can be explored via an online tool: https://burden.sciensano.be/shiny/mortality.

